# Residual efficacy of SumiShield™ 50WG for indoor residual spraying in Ethiopia

**DOI:** 10.1186/s12936-022-04395-0

**Published:** 2022-12-02

**Authors:** Delenasaw Yewhalaw, Eba Alemayehu Simma, Endalew Zemene, Kassahun Zeleke, Teshome Degefa

**Affiliations:** 1 School of Medical Laboratory Sciences, Institute of Health, Jimma University, Jimma, Ethiopia; 2 Tropical and Infectious Diseases Research Center (TIDRC), Jimma University, Jimma, Ethiopia; 3 Departement of Biology, College of Natural Sciences, Jimma University, Jimma, Ethiopia

**Keywords:** SumiShield^™^ 50WG, Actellic 300CS, Indoor residual spraying, Ethiopia

## Abstract

**Background:**

The rate of decay of the biological efficacy of insecticides used for indoor residual spraying (IRS) is an important factor when making decisions on insecticide choice for national malaria control programmes. A key roadblock to IRS programme is insecticide resistance. If resistance is detected to most of the existing insecticides used for IRS (DDT, pyrethroids, organophosphates and carbamates), the logical next choice could be neonicotinoid insecticides, as pyrethroids are used to treat nets. SumiShield^™^ 50WG belongs to the neonicotinoid class of insecticides and has shown promising results in several phase I, II and III trials in different settings. The aim of this study was to assess the persistence of SumiShield^™^ 50WG by spraying on different wall surfaces and determine its decay rates over time in Ethiopia.

**Methods:**

Five huts with different wall surface types (mud, dung, paint and cement) which represented the Ethiopian house wall surfaces were used to evaluate the residual efficacy of SumiShield^™^ 50WG. Actellic 300CS sprayed on similar wall surfaces of another five huts was used as a comparator insecticide and two huts sprayed with water were used as a control. All huts were sprayed uniformly by an experienced spray operator; non-stop starting from the door and moving clockwise to cover the entire wall surface of the hut. The treatments were assigned to huts randomly. The residual efficacy of the insecticide formulations was evaluated against a susceptible insectary-reared population of *Anopheles arabiensis* using WHO cone bioassays.

**Results:**

SumiShield^™^ 50WG resulted in mortality rates of over 80% at 120 h post-exposure on all surface types for up to nine months post-spray, while Actellic 300CS yielded mortality rates of over 80% for eight months after spray.

**Conclusions:**

The results of this trial demonstrated that the residual efficacy of SumiShield^™^ 50WG extends up to nine months on all treated wall surface types. The long-lasting residual efficacy and unique mode of action of the SemiShield^™^ 50WG shows that it could be an ideal product to be considered as a potential candidate insecticide formulation for IRS in malaria endemic countries such as Ethiopia or other sub-Saharan countries where the transmission season lasts up to four months or longer.

**Supplementary Information:**

The online version contains supplementary material available at 10.1186/s12936-022-04395-0.

## Background

The use of vector control in the fight against malaria in sub-Saharan Africa has mostly relied on the massive distribution of long-lasting insecticidal nets (LLINs) and indoor residual spraying (IRS) of insecticides [1–3]. Indoor residual spraying is one of the most effective methods of vector control in settings where mosquitoes are endophilic and endophagic. The benefits of IRS include a strong mass killing effect, no need for continued compliance after the initial spray, unlike bed nets where users need to sleep under the net for it to be effective and some formulations can last for the entire rainy season, providing protection when it is most needed.

Previously, only four classes of insecticide have been recommended for IRS: organochlorines, pyrethroids, carbamates, and organophosphates [4]. In many areas, resistance to organochlorine DDT has been developed by mosquitoes to the point that almost no killing effect is noticed [5–10], even when the spray has been freshly applied [11]. Moreover, the health and environmental issues associated with DDT have restricted its use. Pyrethroids are cheap and long-lasting, but as they are used for net treatment, there are serious concerns about using pyrethroids for IRS when other options are available [4]. This leaves only carbamates and organophosphates as viable alternatives, which is challenging, as these insecticides are more expensive and also share a mechanism (insensitive acetylcholinesterase) conferring resistance to both insecticides [4]. In addition, there is widespread resistance to all the four classes of insecticides [8, 12], suggesting that alternative vector control tools are required to address these challenges. Although other vector control tools such as larval source management are available, and new technologies, such as transgenic mosquitoes, attractive toxic sugar baits and endectocides are under development for reducing malaria transmission [13–16], the use of insecticides remains an essential tool to control endophilic mosquito vectors. Therefore, there is a pressing need to develop new insecticide formulations for IRS, which are effective against mosquito populations that exhibit resistance to the existing classes of insecticides.

In October 2017, a new insecticide formulation, SumiShield^™^ 50WG containing 50% clothianidin, received a prequalification from the World Health Organization (WHO) to be used for IRS to control adult mosquitoes [17, 18]. Clothianidin is a novel neonicotinoid insecticide which acts as agonists of nicotinic acetylcholine receptors within mosquitoes. This novel mode of action gives clothianidin the potential to provide control of vectors in areas of high pyrethroid resistance. An IRS formulation containing clothianidin, SumiShield^™^ 50WG, has been shown to be effective in phase I trials in the Democratic Republic of the Congo and Mozambique [19, 20], phase II trials in Benin and India [21–23], and phase III trials in Tanzania and India [24, 25].

The effectiveness of IRS depends on several factors, including vector resting behavior, residual efficacy of the insecticides, the quality of spraying, and the nature of the sprayed surfaces [26–29]. The residual efficacy of insecticides often varies depending on the type of wall surfaces used for spraying. For instance, a laboratory experiment done in Iran showed that IRS using deltamethrin (K-Othrine WP 5%, target dose: 25 mg ai/m^2^) resulted in at least 80% mortality of *Anopheles stephensi* for 2 months on mud, 4 months on plaster and wood, and 4.5 months on cement wall surface [27]. Djenontin et al.found bendiocarb (WP 80 Ficam, target dose: 400 mg ai/m^2^) to result in at least 80% mortality for 13 weeks on teak wood, 7 weeks on cement, and 6 weeks on red clay [30]. Interestingly, Tangena et al. found bendiocarb (WP 80 Ficam, target dose: 400 mg ai/m^2^) to result in more than 80% mortality for at least 5 months on mud walls, perhaps explained by the fact that the actual applied dose was closer to 1000 mg ai/m^2^ [31]. Etang et al. found nearly 100% mortality for 13 weeks when Ficam WP (target dose 400 mg ai/m^2^) was applied to concrete and wood, but mortality was only 20% after 13 weeks on mud surfaces [29]. Lees et al. found SumiShield^™^ 50WG to kill over 90% of susceptible strain of *An. gambiae* for 18 months, with higher efficacy documented on cement and mud surfaces than wood [32]. The large variation in the results indicates that the type and specific properties of the substrate is important and local testing is necessary to have an accurate expectation of residual efficacy.

As SumiShield^™^ 50WG is now considered for IRS programme in different eco-epidemiological settings, it is important assess the persistence of this insecticide in experimental huts in Ethiopia. Therefore, this study was conducted to determine the residual efficacy of SumiShield^™^ 50WG against susceptible insectary population of *Anopheles arabiensis* strain from Sekoru, and the effect of different wall substrates on the persistence of SumiShield^™^ 50WG in Ethiopia.

## Methods

### Study area and period

The study was conducted in Sekoru district, southwestern Ethiopia at Jimma University Tropical and Infectious Diseases Research Center (TIDRC) from November 2019 to August 2020.

### Experimental hut design

**Fig. 1:**
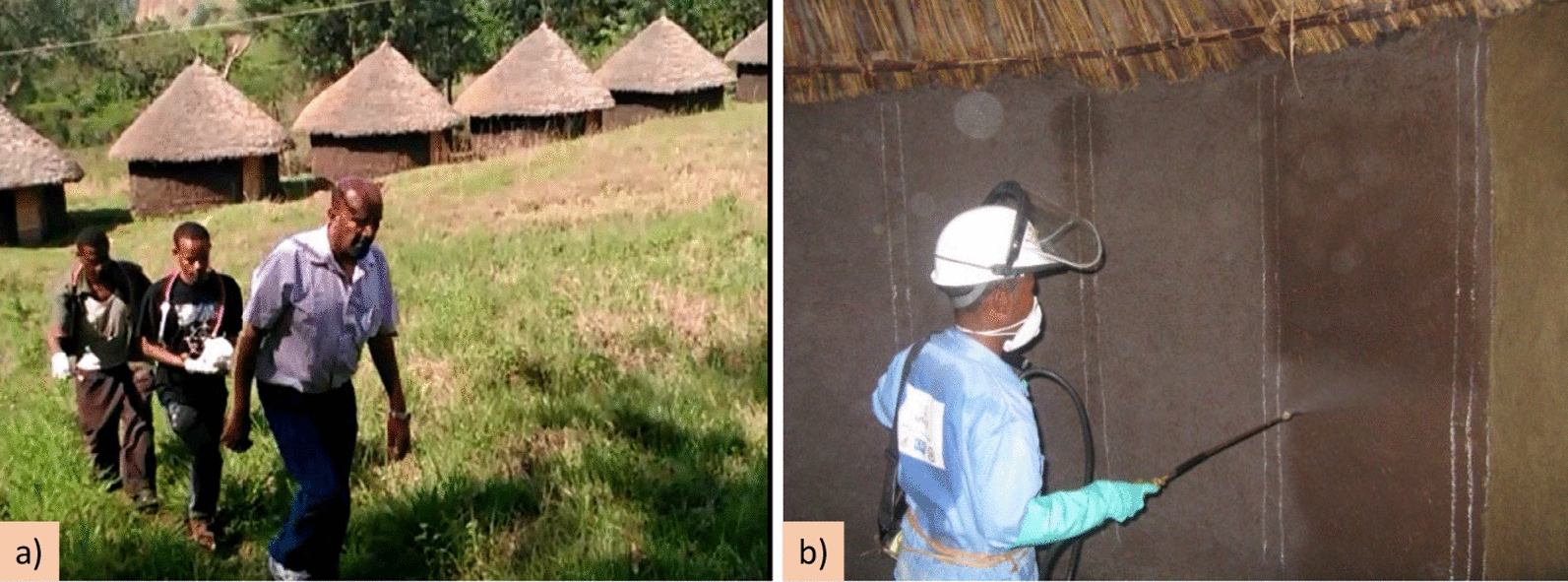
Experimental huts at Tropical and Infectious Diseases Research Center (TIDRC), Jimma University, Ethiopia. **a** the experimental huts with some of the research team members, **b** Spray operator spraying insecticides on the wall surfaces of the hut

Twelve experimental huts constructed by Jimma University TIDRC at Sekoru for evaluation of different insecticide formulations were maintained and used for this trial. The huts were of “tukul” type which were circular huts, constructed using a wattle and daub technique, consisting of a frame of eucalyptus wood, plastered with mud (Fig. 1). The roof was made of a frame of eucalyptus wood beams covered with grass. The interior diameter of the huts was approximately 2.5–3.5 m and the height of the walls was between 2 and 3 m. The interior walls of each experimental hut were plastered with six panels using different materials to make representative of typical wall surfaces of houses of Ethiopia. Each wall surface type in a single hut was then demarcated and labeled as Bako (mud), Cement, Dung, Gambella (mud), Painted and Sekoru (mud). Mud wall surfaces were prepared by collecting soil from three different localities i.e. Bako (low malaria transmission setting), Gambella (high malaria transmission setting), and Sekoru (the study site) to see the effect of different soil types on the residual efficacy of the insecticide formulations. The wall surfaces were prepared one month before insecticide application to dry and allow the pH to stabilize to around 7–8, in order to avoid very high pH levels typically seen in housing with freshly plastered walls.

### Treatments

Three treatments were used in this study: (1) SumiShield^™^ 50WG from Sumitomo Chemical Co., Ltd, (2) Actellic 300CS containing pirimiphos methyl (active ingredient) as a positive control, 3) Water (negative control). Actellic 300CS was selected as a control in this study as it is being used for IRS operation in Ethiopia. Five huts were used to be sprayed with SumiShield^™^ 50WG; another five huts were used to be sprayed with Actellic 300CS, and two huts sprayed with water were used as a control. All huts were sprayed uniformly by the same experienced spray operator; non-stop starting from the door and moving clockwise to cover the entire wall surface of the huts. The treatments were randomly assigned to huts.

### Preparing the spray mixture

SumiShield^™^ 50WG is a water dispersible granule (WG) formulation for IRS to control adult mosquitoes. The active ingredient of this product is clothianidin (50%). SumiShield^™^ 50WG was applied by trained operator with hand-held compression sprayers complying the WHO specifications, fitted with flat fan nozzles (8002E) and a red Control Flow Valve (CFV). The insecticide was mixed according to the instructions on the label. The target dose of the SumiShield^™^ 50WG insecticide is 300 mg ai/m^2^. For the specified target dose, one sachet of SumiShield^™^ 50WG was mixed in 7.5L of water. This was applied to 250 m^2^ of the surface. The volume of spray applied in each experimental hut was determined gravimetrically (by weighing the unpressured sprayer before and after each application in each hut). This allowed calculation of an overall average application rate (target with CFV is 30 ml/m^2^). Prior to initiating treatments, trials with water were conducted to ensure that the operator was consistently able to apply the target dose.

### Assessment of insecticidal spray

TO assess the quality of the spray (doses), 3 filter papers (Whatman No. 1) were fixed on the walls (low, 50 cm from the floor, middle, and high, 50 cm from the junction with the ceiling on the walls) to be sprayed of each hut. These were supported on pins to keep papers clear of the wall surface to prevent them soaking up excess run off of spray. The locations of the papers were marked with colored chalk on wall surfaces to ensure subsequent bioassays were not conducted in locations where papers prevented the spray from touching the walls. The papers were collected 5–6 h after spraying, rolled up in aluminum foil, carefully labelled with date of spray, treatment type, hut number and operator, and stored inside a refrigerator at 4 °C until transported to the laboratory for chemical analysis. The insecticide concentration was analysed using High Performance Liquid Chromatography (HPLC) following the methods previously described [31, 33] at the Quality Control Research Laboratory, Tokyo.

### Mosquito strains

Insectary-reared *Anopheles arabiensis* (Sekoru strain colonized from Adama, Ethiopia) were used for this experiment. This strain was known to be susceptible to all insecticides (DDT, pyrethroids, organophosphates and carbamates). Moreover, susceptibility to clothianidin was also confirmed using impregnated papers before the experiments. Three to five days-old female mosquitoes fed ad libitum with sugar solution (10%) were used for the bioassays. A total of 2,160 female mosquitoes were used for the experiments each month.

### Assessment of residual activity

Standard WHO cone bioassays were conducted on the walls of treated houses for nine months to monitor persistence of the insecticide formulations on sprayed walls. The bioassays were conducted monthly from November 2019 to August 2020 except during the month of June which was missed due to a security issue. Batches of ten female adult mosquitoes from TIDRC insectary were transferred into paper cups covered with netting. Sugar solution-soaked cotton wool was placed on the netting of each cup. Mosquitoes were taken to the experimental huts for the test in a wooden box covered with moist towel to maintain humidity. In each hut and on each surface type, three cones were fixed to walls at different heights (at 50 cm from the junction with the ceiling on the walls (high), middle, and at 50 cm above the floor (low)) of the indoor walls to evaluate the persistence of insecticide at different heights [34]. Cones were attached to the walls using small nails. Then, mosquitoes from each paper cup were transferred into the cones by using a mouth aspirator (a separate aspirator was used for each insecticide formulation). After 30 min of exposure, the mosquitoes were returned to the paper cups with sugar solution on a cotton wool, which were then kept in a wooden box covered with moist towel and mortality was recorded after 24 h and daily for up to day 7. Relative humidity and temperature were recorded during each trial for each experimental hut.

### Data analysis

Data were checked for completeness, consistency and entered into excel sheet. Descriptive analysis was done using the excel data, and then, the excel data were exported in to SPSS version 25 software package for advanced statistical analysis.

Post-exposure knockdown and daily mortality rates over 7 days were reported as mean of the cone test results from five replicate surfaces. When control mortality was between 5 and 20%, experimental mortality was corrected using Abbott’s formula [35], and when mosquito mortality was  > 20% in the control, the result was discarded and the test was repeated. When assessing mortality, the position of the cone on the wall (high, middle or low) was recorded alongside data for each batch of mosquitoes. This allowed correlation of mortality data with each specific part of the hut wall which was particularly useful if one cone was repeatedly giving low activity, as this suggested this area might have been under sprayed. Poison regression model was used to analyse differences in the observed mean mortality between months, the different wall surface types, and height. The residual efficacy of the insecticide formulations was considered satisfactory if the mortality rates were greater than or equal to 80%, in accordance with the WHO criteria [34]. P < 0.05 was considered statistically significant during the analysis.

## Results

### Filter paper data

Filter paper chemical analysis showed that there was an overall average of 415 mg ai/m2 clothianidin and 1,581 mg ai/m2 pirimiphos methyl in SumiShield^™^ 50WG and Actellic 300CS treated wall surfaces, respectively (Additional file 1: Table S1 and Additional file 2: Table S2). This indicates that there was 37–57% overdose for SumiShield^™^ 50WG and Actellic 300CS from their target doses, respectively. Filter papers placed at higher position on the wall surfaces received a dose closer to the target dose for both products (SumiShield^™^ 50WG and Actellic 300CS, 358 mg/m2 and 1,401 mg/m2, or 19% and 41%, above target, respectively).

### Mortality rate

**Fig. 2:**
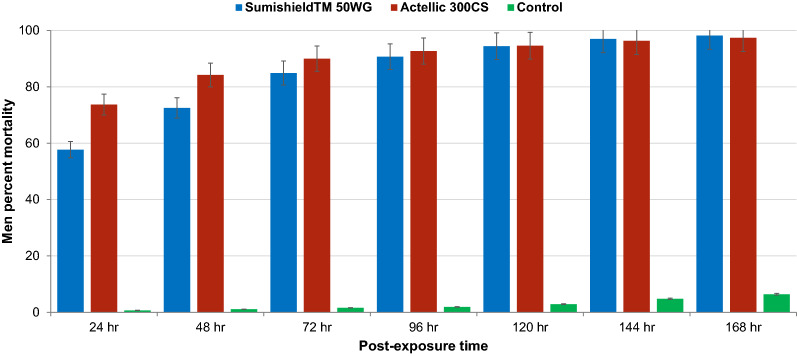
Comparison of the mean percent mortality rates of *Anopheles arabiensis* exposed to SumiShield^™^ 50WG and Actellic 300CS insecticide formulations applied on wall surfaces. The bars represent control corrected percent mortality. The error bars indicate 95% confidence interval

The mean mortality rates of *An. arabiensis* exposed to different wall surface types treated with either SumiShield^™^ 50WG or Actellic 300CS formulations are shown in Fig. 2. Both SumiShield^™^ 50WG and Actellic 300CS yielded a significantly higher mortality rates compared to control. There were no significant differences in mortality rates among the different heights of the wall for both SumiShield^™^ 50WG and Actellic 300CS. Overall, the mortality rates of *An. arabiensis* were significantly higher for Actellic 300CS than SumiShield^™^ 50WG at 24 h (F = 91.965, p < 0.001), 48 h (F = 67.979, p < 0.001), 72 h (F = 19.537, p < 0.001) and 96 h (F = 4.437, p = 0.035) post-exposure. However, no significant difference was documented between the performance of the two insecticide formulations based on 120 h, 144 h and 168 h post-exposure mortality.

On month 0 and during the first two months of the insecticide application (November to January), Actellic 300CS yielded significantly higher 24 h and 48 h mortality rates (p < 0.05) compared to SumiShield^™^ 50WG, whereas SumiShield^™^ 50WG resulted in significantly higher cumulative mortality rates at all times (24–168 h) 9 months after application of the insecticide compared to Actellic 300CS. Moreover, SumiShield^™^ 50WG resulted in a relatively higher 144 h and 168 h post-exposure mortality rates of *An. arabiensis* 8 months after application compared to the Actellic 300CS insecticide formulation (Table 1).

**Table 1:** Monthly mortality rates of *An. arabiensis* after exposure to SumiShield^™^ 50WG and Actellic 300CS formulations applied on different wall surfaces under experimental conditions

Month	Treatment	Monthly percent mortality						
		24 h	48 h	72 h	96 h	120 h	144 h	168 h
0	SumiShield	67.1	81.2	87.7	93.9	99.2	100.0	100.0
	Actellic	99.2	99.7	99.7	99.7	100.0	100.0	100.0
	Control	0	0	0	0	0	0	0
1	SumiShield	56.5	67.1	95.9	100.0	100.0	100.0	100.0
	Actellic	98.2	99.5	100.0	100.0	100.0	100.0	100.0
	Control	0	0	0	0	0	0	0
2	SumiShield	84.5	92.6	98.5	99.5	99.8	100.0	100.0
	Actellic	93.5	97.6	98.6	99.8	100.0	100.0	100.0
	Control	1.7	1.9	1.9	1.9	1.9	1.7	1.9
3	SumiShield	75.8	93.9	98.9	99.1	99.2	100.0	100.0
	Actellic	82.3	92.9	93.8	95.3	95.6	97.1	99.4
	Control	0	0	0.6	0	0	1.7	2.8
4	SumiShield	65.8	84.5	96.4	98.5	99.8	100.0	100.0
	Actellic	78.0	92.1	97.9	99.2	100.0	100.0	100.0
	Control	1.1	0.6	2.5	2.5	2.8	5.0	5.0
5	SumiShield	50.0	63.6	71.4	81.1	85.5	93.5	98.3
	Actellic	83.8	92.1	93.9	95.5	95.8	97.1	98.8
	Control	0.3	0.6	0.8	1.1	4.7	8.1	12.2
6	SumiShield	40.5	47.4	64.5	75.3	83.9	88.2	92.7
	Actellic	44.1	53.9	77.1	84.8	90.9	94.1	97.4
	Control	0.3	0.6	1.1	2.8	5.0	8.1	9.7
8	SumiShield	36.7	50.8	72.9	84.4	93.6	97.4	98.5
	Actellic	54.5	73.3	83.5	87.9	93.0	95.9	97.6
	Control	1.7	2.8	4.2	4.4	5.3	11.1	17.2
9	SumiShield	42.1	70.9	78.0	84.2	88.6	93.5	94.5
	Actellic	29.7	56.7	65.5	72.0	76.2	82.1	83.6
	Control	1.7	3.1	3.6	4.4	6.1	8.1	8.9

**Fig. 3:**
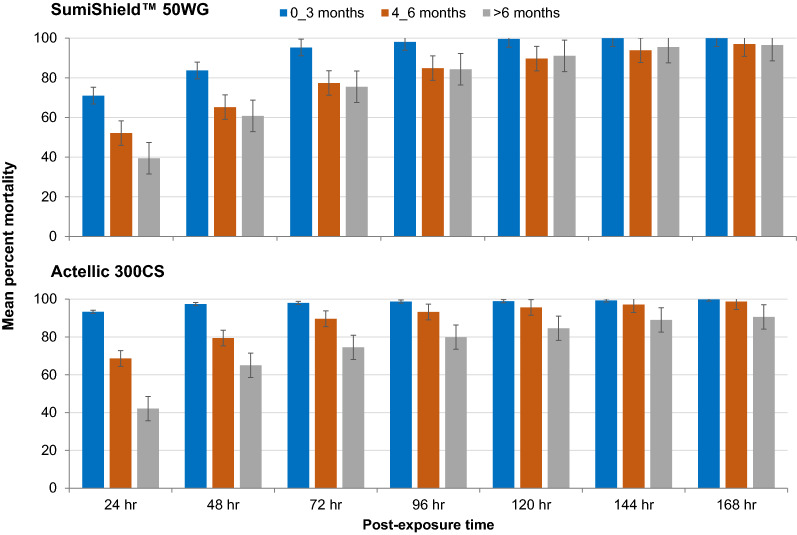
The residual efficacy of SumiShield^™^ 50WG and Actellic 300CS averaged across different month intervals after application of the insecticides on wall surfaces. The bars represent control corrected percent mortality. The error bars indicate 95% confidence interval

The residual efficacy of the SumiShield^™^ 50WG insecticide formulation varied significantly between months (p < 0.05). The mean 24 h mortality was 71% during the first three months (November–February) of the insecticide application, and declined to 52.1% during the 4th–6th months (March to May), and to 39.4% during the 8th to 9th months (July to August). The mean mortality rates at 120–168 h post exposure were over 80% for up to 9 months after application of the insecticide (Table 1, Fig. 3).

Similarly, the residual efficacy of Actellic 300CS insecticide formulation varied significantly between months (p < 0.05). The mean 24 h mortality was 93.3% during the first three months (November–February) following the insecticide application, and declined to 68.6% during the 4th–6th months (March to May), and to 42.1% during the 8th to 9th months (July to August). The cumulative 48 h and 72 h mortality rates were above 92% from month 0 to month 5 after which it started to decline to below 80%. The mean 120–168 h mortality rates were over 80% for up to 8 months after application of the insecticide (Table 1).

### Effect of wall surface type

**Fig. 4:**
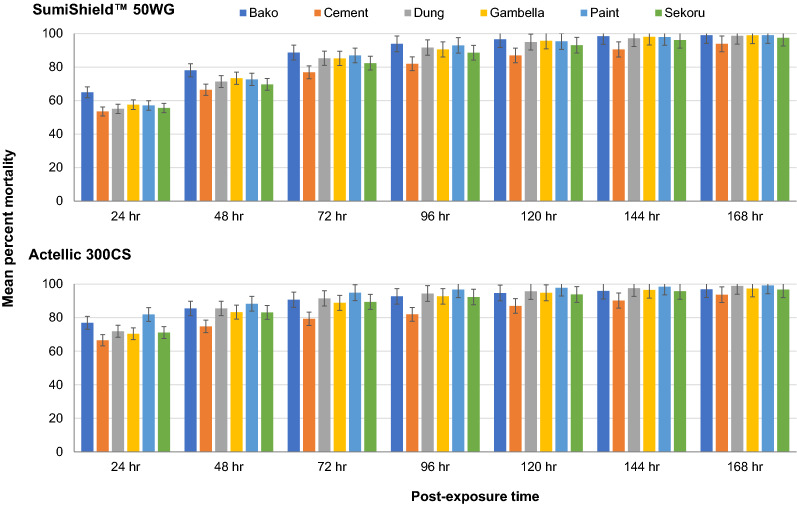
Comparison of the mean percent mortality rates of *Anopheles arabiensis* exposed to SumiShield^™^ 50WG and Actellic 300CS insecticide on six different wall surfaces. The bars represent control corrected percent mortality. The error bars indicate 95% confidence interval

For SumiShield^™^ 50WG, the mean 24 h and 48 h mortality rates showed significant variation between the different wall surface types (p < 0.05). Mud (Bako) wall surface yielded the highest mortality rate, while cement wall surface yielded the lowest mortality rate (Table 2, Fig. 4). However, no significant differences in mortality rates were documented among the wall surface types at longer holding periods (72–168 h post-exposure) (p > 0.05).

**Table 2:** Mean percent mortality rates of *An. arabiensis* exposed to different wall surfaces treated with SumiShield^™^ 50WGand Actellic 300CS (December 2019-August 2020)

Surface type	Treatment type	Monthly percent mortality						
		24 h	48 h	72 h	96 h	120 h	144 h	168 h
Bako (mud)	SumiShield	65.0	78.1	88.7	93.9	96.6	98.5	99.1
	Actellic	76.9	85.5	90.7	92.7	94.7	95.9	96.9
	Control	0.2	0.7	1.7	2.0	3.0	4.1	6.5
Cement	SumiShield	53.5	66.5	76.9	82.0	87.0	90.6	93.9
	Actellic	66.5	74.8	79.3	82.0	87.0	90.2	93.7
	Control	0.7	1.1	1.5	2.2	3.1	6.3	8.9
Dung	SumiShield	55.1	71.4	85.3	91.7	95.0	97.2	98.7
	Actellic	71.9	85.5	91.5	94.4	95.7	97.6	98.9
	Control	1.1	1.3	1.9	1.7	2.0	4.1	5.0
Gambella (mud)	SumiShield	57.6	73.4	85.2	90.6	95.7	98.1	99.0
	Actellic	70.4	83.3	88.8	92.7	94.8	96.5	97.3
	Control	0.9	1.1	1.3	1.7	3.1	4.6	5.7
Paint	SumiShield	57.1	72.7	87.0	93.0	95.4	98.0	99.1
	Actellic	81.9	88.3	94.9	96.8	97.8	98.5	99.2
	Control	0.6	0.9	1.3	1.3	2.2	4.3	5.2
Sekoru (mud)	SumiShield	55.6	69.7	82.4	88.6	93.1	96.2	97.5
	Actellic	71.1	83.1	89.4	92.3	93.8	95.8	96.8
	Control	0.9	1.7	2.2	2.6	3.7	5.7	7.2

For Actellic 300CS, the mean 24 h mortality rate showed significant variation among the different wall surface types (p = 0.003). The painted wall surface yielded the highest mortality rate in almost all months, followed by dung and mud (Bako) wall surface types. Similar to SumiShield^™^ 50WG, the lowest mortality rates were documented for Actellic 300CS applied on the cement wall surface as compared to the other wall surface types (Fig. 4).

## Discussion

Given the widespread resistance of malaria vectors to commonly available insecticides, there is a pressing need to develop and/or evaluate new or alternative chemical insecticides with different modes of action to enhance the control of resistant vector populations [36]. The aim of this study was to evaluate the residual efficacy of SumiShield^™^ 50WG containing the insecticide clothianidin sprayed on different wall surface types in Ethiopia. The findings of this study showed that the 120 h mortality of *An. arabiensis* exposed to SumiShield^™^ 50WG exceeded 80% for up to nine months after spray. This shows that the residual efficacy of SumiShield^™^ 50WG extends up to nine months which would appear to be suitable for Ethiopian malaria transmission season which lasts for at least four months mainly from September to December.

During the first two months following insecticide application, Actellic 300CS yielded significantly higher 24 h and 48 h mortality rates compared to SumiShield^™^ 50WG. This difference could be due to higher overdosing of Actellic 300CS compared to SumiShield^™^ 50WG. In this trial, an overdose of 57% from the target application rate was documented for Actellic which is above the limit recommended by WHO, while this was 37% for SumiShield^™^ which was within  ± 50% of the target dose recommended by the WHO [37]. This might have overestimated the residual efficacy of Actellic formulation in this study. Moreover, the slower acting nature of clothianidin, the active ingredient of SumiShield^™^ 50WG [20, 32], could be another factor for the lower mortality rates of SumiShield^™^ 50WG compared to Actellic 300CS at the 24 h and 48 h holding periods.

Although lower mortality rates were observed at shorter holder periods (24–48 h), SumiShield^™^ 50WG is shown to be effective in killing over 80% of mosquitoes after longer holding periods (120-168 h) for nine months post-spray, which is comparable to Actellic 300CS which also resulted in mortality rates of over 80% for eight months post-spray. This suggests that both SumiShield^™^ 50WG and Actellic 300CS could be effective for IRS operation to be used in malaria endemic sub-Saharan African settings where malaria transmission occurs throughout the year [38–40]. The long residual efficacy of SumiShield^™^ 50WG coupled with its unique mode of action, which reduces the probability of selecting for insecticide resistance [41], suggests that it could be a product of choice for IRS operation to control malaria vectors in Ethiopia.

Several studies have also documented the optimal residual efficacy of over six months for SumiShield^™^ 50WG in other studies conducted elsewhere [19, 24, 32]. In the Democratic Republic of Congo for instance, SumiShield^™^ 50WG was shown to result in mortality rates of over 80% for more than nine months [19]. In Tanzania, SumiShield^™^ 50WG maintained optimal efficacy in field settings for the duration of 6-month period, with 100% mortality of mosquitoes by 144 to 168 h post-exposure to treated surfaces [24]. In Mozambique, SumiShield™ 50WG was found to be efficacious for 6.5 to 9.5 months based on 72 h mortality post-exposure [20]. In a study conducted by Lees et al. SumiShield^™^ 50WG was shown to be effective against both insecticide resistant and susceptible strains of *Anopheles gambiae* and *Anopheles funestus* for up to 18 months [32]. In the current study, the residual efficacy of SumiShield^™^ 50WG was monitored for up to nine months only, with over 80% mortality rates documented by 120 h and over 92% by 168 h during the nine months period. It is therefore possible that that the efficacy of SumiShield^™^ could extend beyond nine months if its efficacy was monitored for longer duration.

In this study, the residual efficacy of SumiShield^™^ 50WG varied significantly between different wall surface types at 24 and 48 h post-exposure. The highest mortality rate was documented on mud (Bako) wall surface followed by paint wall surface. However, there were no significant differences among the wall surface types in terms of delayed mortality (96 to 168 h). For Actellic 300CS on the other hand, the highest mortality rates were recorded from painted wall surface in almost all months, followed by dung and mud (Bako) wall surfaces. In contrast, lowest mortality rates for both SumiShield^™^ 50WG and Actellic 300CS insecticide formulations were recorded from cement wall surface type. Such differences in residual efficacy of insecticides between different wall surface types have also been documented in several studies [19, 20, 32].

In conclusion, the residual efficacy of SumiShield^™^ 50WG extends up to nine months, which would appear to be suitable for Ethiopia main malaria transmission season that lasts at least for four months mainly occurring from September to December. The long-lasting residual efficacy and unique mode of action of SemiShield^™^ 50WG suggests that it could be an ideal product to be considered as a potential candidate insecticide formulation for IRS in malaria endemic countries.

## Supplementary Information

**Additional file 1: Table S1.** Clothianidin contents on filter papers sprayed with SumiShield^TM^ 50WG on different wall surfaces of experimental huts, Ethiopia (2020).

**Additional file 2: Table S2.** Pirimiphos-methyl contents on filter papers sprayed with Actellic 300CS at different heights of wall surfaces of the experimental huts, Ethiopia (2020).

## Data Availability

Data supporting the conclusions of this article are included within the article. Raw data are available from the corresponding author upon reasonable request.

## Abbreviations

CFV: Control flow valve
IRS: Indoor residual spraying
LLIN: Long lasting insecticidal net
TIDRC: Tropical and infectious diseases research center
WG: Water dispersible granule
WHO: World Health Organization
WP: Wettable powder
