# Supplementary material for: Residual efficacy of SumiShield™ 50WG for indoor residual spraying in Ethiopia
Source: Malar J. 2022 Dec 2;21:364. doi: 10.1186/s12936-022-04395-0 (PMC9716761; doi:10.1186/s12936-022-04395-0)
Supplement: Supplementary file 1 — Additional file 1: Table S1. Clothianidin contents on filter papers sprayed with SumiShieldTM 50WG on different wall surfaces of experimental huts, Ethiopia (2020). [file 12936_2022_4395_MOESM1_ESM.docx]

Table S1: Clothianidin contents on filter papers sprayed with SumiShield^TM^ 50WG on different wall surfaces of experimental huts, Ethiopia (2020)

| Sample ID* | Wall material | Vertical position on the wall – clothianidin (mg/m^2^) | | | |
| --- | --- | --- | --- | --- | --- |
|  |  | Low | Middle | High | Mean |
| C1 | Cement | 430 | 415 | 238 | 361 |
| C2 | Cement | 371 | 823 | 438 | 544 |
| H1 S | Sekoru/Mud | 518 | 528 | 43 | 363 |
| H1 D | Dung | 197 | 348 | 413 | 319 |
| H1 P | Paint | 336 | 504 | 517 | 452 |
| H1 B | Bako/Mud | 220 | 716 | 587 | 508 |
| H1 G | Gambella/Mud | 393 | 558 | 331 | 427 |
| H2 D | Sekoru/Mud | 490 | 539 | 667 | 565 |
| H2 P | Dung | 310 | 411 | 536 | 419 |
| H2 B | Paint | 293 | 447 | 332 | 357 |
| H2 G | Bako/Mud | 245 | 298 | 175 | 239 |
| H2 S | Gambella/Mud | 166 | 383 | 356 | 302 |
| H3 S | Sekoru/Mud | 392 | 446 | 228 | 355 |
| H3 D | Dung | 281 | 403 | 344 | 343 |
| H3 P | Paint | 334 | 708 | 345 | 462 |
| H3 B | Bako/Mud | 311 | 353 | 275 | 313 |
| H3 G | Gambella/Mud | 322 | 514 | 309 | 382 |
| H4 S | Sekoru/Mud | 478 | 485 | 337 | 433 |
| H4 D | Dung | 629 | 661 | 418 | 569 |
| H4 P | Paint | 425 | 503 | 294 | 408 |
| H4 B | Bako/Mud | 627 | 698 | 266 | 530 |
| H4 G | Gambella/Mud | 351 | 646 | 433 | 477 |
| Mean |  | 369 | 518 | 358 | 415 |

* H = Experimental hut number. C1 and C2 are separate cement huts

Table S2: Pirimiphos-methyl contents on filter papers sprayed with Actellic 300CS at different heights of wall surfaces of the experimental huts, Ethiopia (2020)

| Sample ID* | Wall material | Vertical position on the wall – pirimiphos-methyl (mg/m^2^) | | | |
| --- | --- | --- | --- | --- | --- |
|  |  | Low | Middle | High | Mean |
| C1 | Cement | 2011 | 1649 | 1057 | 1572 |
| C2 | Cement | 1545 | 2211 | 1676 | 1811 |
| H5 S | Sekoru/Mud | 1076 | 1439 | 1010 | 1175 |
| H5 B | Bako/Mud | 1276 | 1677 | 1086 | 1346 |
| H5 P | Paint | 1495 | 1304 | 1148 | 1315 |
| H5 D | Dung | 1915 | 1630 | 1257 | 1601 |
| H5 G | Gambella/Mud | 1423 | 1408 | 879 | 1236 |
| H6 S | Sekoru/Mud | 1520 | 1301 | 1205 | 1342 |
| H6 B | Bako/Mud | 1056 | 1240 | 809 | 1035 |
| H6 P | Paint | 1772 | 2620 | 2208 | 2200 |
| H6 D | Dung | 1876 | 1740 | 1414 | 1677 |
| H6 G | Gambella/Mud | 1202 | 1277 | 1015 | 1165 |
| H7 S | Sekoru/Mud | 1225 | 1259 | 1395 | 1293 |
| H7 B | Bako/Mud | 1462 | 1673 | 1293 | 1476 |
| H7 P | Paint | 2054 | 2986 | 2207 | 2416 |
| H7 D | Dung | 1242 | 2265 | 1539 | 1682 |
| H7 G | Gambella/Mud | 1442 | 1535 | 1122 | 1366 |
| H8 S | Sekoru/Mud | 2347 | 2175 | 2025 | 2183 |
| H8 B | Bako/Mud | 1583 | 1767 | 1608 | 1653 |
| H8 P | Paint | 2033 | 2935 | 1946 | 2305 |
| H8 D | Dung | 1364 | 1711 | 2026 | 1700 |
| H8 G | Gambella/Mud | 1438 | 1389 | 909 | 1245 |
| Mean |  | 1562 | 1781 | 1401 | 1581 |

* H = Experimental hut number. C1 and C2 are separate cement huts
